# Reinforced Polymer Composites III

**DOI:** 10.3390/polym15092069

**Published:** 2023-04-27

**Authors:** Victor V. Tcherdyntsev

**Affiliations:** Laboratory of Functional Polymer Materials, National University of Science and Technology “MISIS”, Leninskii Prosp, 4, 119049 Moscow, Russia; vvch@misis.ru; Tel.: +7-9104002369

The development of modern technology requires the development of new materials with improved operational and technological properties. Many of the currently known natural and artificial materials no longer meet the increasingly high requirements. The discovery of fundamentally new materials is an extremely rare phenomenon, which indicates that the overwhelming majority of “simple” materials have already been discovered, and we should not expect great achievements in this regard. Therefore, the main direction in the development of new materials is now the creation and improvement of composite materials.

Depending on the intended application of composites, polymers can be reinforced by both dispersed (particles) and continuous (fibers and fabrics) fillers. When developing a new composite material, many factors must be considered. Particular attention should be paid to operating temperatures, which largely determine the choice of polymer matrix. To effectively select suitable candidate materials, first, one needs to apply search filters to the critical properties.

For instance, if the developed composites are intended for tribological application, the following critical properties should be considered: the PV factor (dry friction, in water, and boundary lubrication), continuous operating temperature, heat resistance, and coefficient of thermal expansion. A significant increase in the thermal conductivity of a polymer-based composite is achieved with a filling of 50 vol. % and above. When the filling is less than 40 vol. %, the thermal conductivity of the composite, as a rule, remains at a sufficiently low level in order to significantly improve the level of heat removal from the tribocoupling zone. Therefore, optimizing the composition of a composite in terms of thermal conductivity is ineffective if this simultaneously worsens its ductility and crack resistance. A more effective solution is to choose a high-temperature polymer matrix that can withstand temperature changes without changing the working gaps and the loss of the load capacity of the composite, as well as ensuring low friction losses to minimize the frictional heating of the contact surfaces.

The choice of dispersed fillers is determined by the particle size and particle size distribution. The efficiency of dispersed fillers depends on the specific surface of the particles, especially in cases in which surfactants, dispersing agents, surface modifiers, etc., are adsorbed or interact on the surface of the filler. The nature of the packing of filler particles is essential, especially to obtain highly filled compositions. Usually, different fractions of filler are mixed to achieve the minimum free volume. Regarding the chemistry of the surface of filler particles, the free surface energy (surface tension) is of primary importance, which determines the adhesive characteristics of the filler surface and its wettability. The presence of certain functional groups on the surface of a filler and their reactivity is also very important.

The next step in creating a composite is the development of ways to introduce a filler into the polymer matrix. The problem of filler distribution is as important as the problem of matrix–filler boundary adhesion. Particles (especially nanosized particles) have a high tendency to agglomerate, which can lead to the appearance of weakened regions in the composite, zones of increased stress, and defect concentrations, and this can also reduce the efficiency of nanoparticles. Therefore, it is important to develop an effective technique for mixing fillers with a polymer matrix.

When developing composites, there are two fundamental problems, the degree of the solution of which will determine the final properties of the created materials:

– Ensuring the uniform distribution of the filler over the volume of the matrix;
– Ensuring the high adhesion of fillers with a polymer matrix.

Fiber-reinforced composites are another class of polymer composites. Thermosetting polymers are the most widely used today as matrix materials for composites reinforced with continuous fibers and fabrics. Prepregs for forming composites based on thermosetting polymers are sensitive to temperature and must be stored in a refrigerator to extend their shelf life; otherwise, the curing reaction may start prematurely, making the material unsuitable for further processing. At the same time, thermoplastic polymers do not require any additional chemical reactions to transform the starting materials into the final product. The compaction of such materials is achieved by a simple heating process due to the melting and subsequent cooling of the material under a certain level of contact pressure. The shelf life of these materials under normal conditions is unlimited, and there are no special requirements for their storage. Thermoplastic matrix composites are stiffer and less brittle than thermoset composites, with very good impact strength and damage resistance. Thermoplastic polymers tend to have lower densities, making them a viable alternative for applications with stringent weight and size requirements. The most important aspect is that the processes of crystallization (or glass transition) are reversible; therefore, products made from such materials are repairable, and their shape and dimensions can be changed simply by heating them. In addition, this greatly simplifies the recycling process of such products. Even if it is not possible to remove the polymer matrix, it is possible to grind such materials, obtain granules from them, and use the granules to obtain products reinforced with discrete, randomly oriented fibers, through, for example, injection molding or extrusion.

Composite materials reinforced with fibers are used in many branches of engineering due to their good specific mechanical characteristics. Exploratory research to further improve the performance of such materials follows several paths. One of these paths is the joint use of different fibers in the composition of one composite, which makes it possible to reduce the cost of such materials while maintaining the required level of mechanical properties. Another path aims toward increasing the level of mechanical, tribological, physical, and other properties of fiber-reinforced composites. The implementation of this path is mainly carried out due to the introduction of dispersed particles, mainly nanosized ones, into the compositions of such composites. Particles can either be applied to the surface of reinforcing fibers or introduced into the bulk of a polymer matrix.

This Special Issue covers both aforementioned types of reinforced polymer composites and consists of 17 research articles. Zerbinati et al. [1] reported the application of polyethylene glycol diglycidyl ether as a cross-linker to increase the mechanical behavior of injectable hyaluronic acid dermal fillers. It was shown that cross-linking allows hyaluronic acid hydrogels’ complex viscosity and resistance to elongation to increase by more than ten times, and the addition of a small amount of hydroxyapatite results in a further increase in the magnitude of the complex viscosity. The analysis of the obtained composite hydrogels showed that cross-linked dermal fillers have fibrous “spiderweb-like” matrix structures, representing fibrous/porous networks with different levels of homogeneity; this feature of spiderweb organization is closely related to monophasic gels.

In addition to the above-mentioned paper, Fouly et al. [2] analyzed the effect of a small amount of hydroxyapatite on the behavior of poly(methyl methacrylate). It was observed that the introduction of 0.8 wt. % of hydroxyapatite in poly(methyl methacrylate) increased the compressive yield stress and Young’s modulus by 1.3 and 1.6 times, respectively, in relation to pure polymers. A tribological study showed that the wear of the obtained composites decreased by 1.5 to 2.0 times, whereas the friction coefficient decreased by 20% with respect to unfilled poly(methyl methacrylate). The composites that were developed are considered to have probable applications as denture base materials.

Winkler et al. [3] considered the orientation of short (0.2 to 10 mm) carbon fibers coated with metal layers in a melted polymer matrix under the influence of an applied magnetic field. An advanced simulation model was created to realize a simulative representation and reveal the fluid–structure interaction, as well as the influences of these parameters on the inducible magnetic torque and fiber alignment of a single fiber. The effects of various parameters, such as melt viscosity, the relative permeability of the coating, coating thickness, fiber length, and magnetic flux density, were determined. The simulation showed that alignment process velocity increases with increases in coating permeability, coating thickness, and magnetic flux density, whereas increases in fiber length and melt viscosity result in a decrease in alignment rate. It was observed that the magnetic flux density and the coating material have the greatest influences on fiber alignment.

Another aspect of the application of short carbon fibers in polymer composites was investigated in [4]. In this study, nitrile butadiene rubber was reinforced with shungite and chopped carbon fibers. Composite samples were carbonized under various regimes, and the final carbonization temperature varied from 280 to 380 °C. A three-point bending test was used to evaluate the crack resistance of the materials obtained. The variation in the stress intensity factor K_1c_ values obtained ranged from 1 to 5 MPa·m^1/2^, which are values that are most typical for brittle materials such as graphite or ceramics. The K_1c_ value significantly depended on the maximum carbonization temperature; the highest values were achieved in compositions carbonized at a lower temperature (280 °C). The addition of short carbon fibers to the composite material did not significantly increase the crack resistance. However, carbon fibers strongly affected the crack propagation process,. While in samples containing no carbon fibers, the crack exhibited almost straight propagation, in the case of carbon-fiber-reinforced composites, at a certain moment of propagation, the crack turned at almost a right angle.

The determinative influence of interaction at the polymer–filler interface on the functional properties of a composite was clearly highlighted in [5]. Polytetrafluoroethylene-based composites reinforced with Zr-based metallic glass powder were prepared using the ball milling technique. The treatment of metal glass surfaces with triethoxyvinylsilane improved the adhesion between the surfaces of the metallic glass and the polymer. The formation of interlinked bridges between the metallic glass and polymer matrix provided via the formation of Si-O-Si bonds between the polymer and filler was observed as a result of silane treatment. The thermal conductivity of the obtained composites increased by 1.2 to 2.2 times in relation to those obtained using no silane treatment. It should be noted that the thermal conductivity value for the obtained composites was not so high because the thermal conductivity of the Zr-based metal glass was low enough. Nevertheless, the data obtained indicate this is an effective method of improving the functional properties of polymer composites.

Generally, thermally conductive polymer composites are of particular interest for researchers. Yu et al. [6] reported the formation of silicon-rubber-based composites filled with boron nitride. Two types of hexagonal BNs differing in their particle shape (2D-flake-like or spherical) were used as fillers, and the filling degree of the composites was up to 40 wt. %. To overcome the problem of high viscosity, which prevents the homogeneous distribution of filler in the polymer matrix, the authors suggested a new “hypergravity accumulation” strategy consisting of the simultaneous centrifugation of raw silicon rubber and boron nitride. Such a strategy has been proven to be an effective method to achieve composites with homogeneous structures, as the thermal conductivity increased by more than two times in relation to the same composites obtained using no centrifugation. The high filling degree of composites coupled with centrifugation treatment enabled the achievement of thermal conductivity values up to 4.0 W/m·K. The interesting result of this study is the linear dependence that was revealed between the thermal conductivity of the composites and the relative gravity acceleration at centrifugation. One more important note is that composites filled with boron nitride with spherical particle shapes possessed higher thermal conductivity levels that those filled with particles with flake-like shapes. The authors associated this unexpected result with the fact that the silicon-rubber-based mixture containing spherical boron nitride had less of a high-viscosity problem than those containing flake-like boron nitride particles.

As well as in the research mentioned above, in [7], the effect of the shape of the filler particles on the thermal conductivity of composites was investigated. Three types of graphite fillers, namely, artificial graphite with nearly equiaxial particles, natural graphite with flakes-like particles, and thermoexpanded graphite with particles representing a set of exfoliated flakes, were used. Polysulfone was used as the matrix material to prepare composites with the polymer solution technology, which avoids high-viscosity problems. The highest values of thermal conductivity were achieved for composites containing natural graphite; for composites containing 70 wt. % of filler, the magnitude was 4.2 W/m·K. Studying the morphology and structure allowed the authors to conclude that natural graphite has a perfect crystalline graphite structure, which makes it the best filler type in terms of its conductive properties. As in the case of solution technology, the abovementioned high-viscosity problems did not occur, and the flake-like particles could freely form an effective conductive net in the polymer matrix, whereas the nearly spherical shape of artificial graphite only allowed point contacts to be created between filling particles. 

Tomiak et al. [8] also used various types of graphite, such as “simply” graphite and expandable graphite, as a filler for polymer composites, and polyamide 6 was used as the matrix material. In this case, a twin-screw compounder (co-rotating) with further injection molding was used to prepare the composites. Unfortunately, the authors provided no data on the initial graphite particles’ shapes. However, it was observed that peculiarities in the injection process resulted in the orientation of “simply” graphite particles along the injection axe, which allowed the authors to propose that “simply” graphite particles have flake-like shapes. In this study, nearly no effect of the filler type on the thermal conductivity value was observed, probably because expandable graphite has the same particle shape as “simply” graphite. By filling polyamide 6 with graphite up to a filling degree of 70 wt. %, composites with thermal conductivity of about 9.1 W/m·K were obtained. However, the type of graphite filler strongly affected the flame retardance behavior of the composites. V0 classification (burning stops within 10 s on a vertical specimen; drips of particles are allowed as long as they are not inflamed) could be achieved in expandable-graphite-filled composites at a filling degree of 25 wt. %, whereas for composites filled with “simply” graphite, this level could only be achieved at a filling degree of 70 wt. %.

One more paper by the same research team [9] reported the flame retardance properties of short-glass-fiber-reinforced polyamide 6 filled with expandable graphite, aluminum diethylphosphinate, and melamine polyphosphate. It was noted that surface-modified glass fibers were used in the study, but neither the details of such a modification nor the effect of the modification on the reinforcers to polymer adhesion were provided. Here, the method of composite formation was the same as that in [8]. It was observed that glass fiber reinforcement changes the expansion behavior of expandable graphite particles and thus reduces the flame-retarding effects; therefore, V0 classification was only achieved for samples containing no glass fibers. However, it was found that the char residue characteristics of glass-fiber-reinforced systems containing expandable graphite particles were superior in terms of stability, withstanding impressive manual impact forces. The authors noted that this might be of particular importance for some applications that require long-term residue stability even under certain physical stress levels while providing good long-term properties, inhibiting the spread of flames.

Eskenati et al. [10,11] studied the mechanical behavior of the glass-fiber-reinforced polymer I-shape pultruded profile-based structures. In [10], the profiles formed of E-glass non-continuous fibers embedded into an isophthalic polyester resin matrix were studied. Flange connectors to connect profile pieces were prepared with epoxy resin reinforced with unidirectional carbon fibers. Two types of connections, namely, bolted and adhesive, were investigated. The experimental study of the mechanical behavior of such a structure was realized together with a numerical simulation. It was observed that bolted connections achieved greater load-bearing capacities than adhesive ones. Adhesive connections are always associated with fragile debonding failure types. The comparison of web-only joints with the web and flange joint showed that the inclusion of a flange connection redistributed stresses in the joint, promoted a more uniform joint response, and unloaded the web connector. Flange connectors were shown to be more effective for larger angles between profiles, because the mechanical stiffness of flange connectors increased when the loading configuration did not increase the initially existing curvature of the fibers. It was found that flange connection increased the joint stiffness by 7.6 times. In [11], glass-fiber-reinforced profiles based on the polymeric matrix of unsaturated polyester and thermosetting were studied. Profile pieces were connected to each other with bolted joints using the connected plates extracted from the web of the same profile. The structure made of connected profiles was jointed with plates made of fabric-reinforced cementitious matrix composites, formed by Portland-cement-based mortar filled with fiber glass mesh. The behavior of the obtained hybrid superficial elements was investigated via both mechanical tests and numerical studies. These investigations showed that hybrid superficial elements behaved linearly up to initial mortar cracking, and after that, the fabric-reinforced cementitious matrix behaved as a plate with no linear response up to the flexural mortar failure. It was found that increasing the thickness of the mortar matrix of the fabric-reinforced cementitious matrix plate had a significant impact on the load-bearing capacity of the hybrid superficial elements studied.

Song et al. [12] considered the aerodynamic and acoustic behavior of rotors for unmanned aerial vehicles created via 3D printing using the bionic edge design approach as an alternative to commercial rotors made of carbon-fiber-reinforced polymers. The authors used polyamide PA 12 and Resin 9400 polymers to form rotors with various edge designs. Better aerodynamic properties were observed for rotors that copied the combination of both the leading and trailing edges of an owl’s wing. Additionally, it was noted that polyamide with better toughness could absorb slight tremors, resulting in less noise. The main problem indicated by the authors was that the unfilled polymers used, which were available for 3D printing, did not provide the required level of the mechanical properties of the rotors. The authors highlighted that although carbon-fiber-reinforced polymers seem to be a promising choice to manufacture bionic rotors with competitive mechanical properties, it is difficult to manufacture rotors with satisfactory surface qualities via 3D printing. This indicates the importance of future development in the field of fiber-reinforced polymers for 3D printing.

The article in [13] reported the study of twill-weave-carbon-fabric-reinforced polyethersulfone-based composites prepared using the polymer solution technique. It was shown that the surface modification of carbon fabrics via thermal oxidation significantly increased the mechanical properties of the composites. The flexural strength and elastic modulus of the composites reinforced with modified fibers reached approximately 962 MPa and 60 GPa, respectively, compared with values of approximately 600 MPa and 50 GPa reached for the composites reinforced with the initial fibers. The study of thermal conductivity showed that the obtained composites realized the thermal conductive properties of carbon fibers at a relatively high level, up to 55–60%. The study of thermal expansion showed that in the direction parallel to the axis of reinforcement, the coefficient of thermal expansion became negative, because carbon fibers shrink along their axis with an increase in temperature. Such results show that it is potentially possible to develop and obtain composite materials with a particular fiber content in which the coefficient of thermal expansion is close to zero in the investigated range of temperatures.

In the study by Dessalegn et al. [14], polypropylene-based composites reinforced with bamboo fibers were prepared via hot pressing. Fibers produced from various varieties and plant regions of bamboo were compared. Additionally, the effect of the harvesting month on the bamboo fibers’ mechanical properties was studied, and it was observed that the harvesting month of November produced better mechanical properties compared to February. The tensile test of the composites showed the Young’s modulus value of up 25 GPa and the ultimate tensile strength of up to 125 MPa.

The article by Hosen and coauthors [15] reported the study of steel fiber content on the mechanical behavior of concrete composites. Portland cement was used as the base of such composites, and artificial mining sand as well as palm oil clinker powders and aggregates were used as fillers; additionally, a polycarboxylate-ether-based additive was utilized as a superplasticizer. It was found that the addition of steel fibers increased the compressive strength of such composites by 20% and that the flexural strength increased by almost four times. The elastic modulus of the steel-fiber-reinforced composites was 1.5 times higher than that of the unfilled ones. The compression and displacement ductility increased by 5–6 and 3 times, respectively, as a result of steel fiber reinforcement.

Another kind of cement-based composite was considered by Yu et al. [16]. A matrix of such cemented past backfill composites was formed using Portland cement and tailings sourced from an iron mine. Two types of polymer fibers, namely, recycled tire polymer fiber, consisting of polyester with the impurity of rubber, and commercial polypropylene fiber, were used as fillers. Mechanical tests showed that the addition of fibers up to 0.6 wt. % significantly increased the failure strain, ductility index, unconfined compressive strength, and toughness of the concrete. The increase in failure strain, ductility index, and unconfined compressive strength was higher for composites filled with recycled tire polymer fibers. When the fiber content increased up to 0.9 wt. %, composites filled with commercial polypropylene fiber maintained the high level of mechanical properties, whereas in the case of recycled tire polymer fiber filler, the mechanical properties drastically fell at such fiber content levels. The ordinary cemented past backfills showed brittle failure with wide major cracks and falling blocks, while the polymer-fiber-reinforced cemented past backfills showed a bulging failure mode with several small cracks, so it could remain integrated under a large strain.

Finally, Sapiai et al. [17] reported a mechanical property study of polyurethane-based hybrid multilevel composites. Twill weave basalt fibers and chopped strand mat glass fibers were used as reinforcers. Additionally, the polymer matrix was filled with granite dust. The effect of the granite dust content on the composite behavior was investigated. The unhole tensile test showed that the addition of granite dust had nearly no effect on the tensile strength and elastic modulus, whereas the tensile strength at break decreased with the increase in granite dust content. The strength reduction value, representing the ratio of tensile strength in the hole and unhole tests, significantly decreased with an increase in granite dust content. The investigated composites showed brittle fracture behavior, whereby delamination mode failure occurred due to fiber breakage and matrix cracking. Flexural and interlaminar shear strength tests showed that the addition of 1 wt. % of granite dust into the polyurethane matrix resulted in significant increases in the elastic modulus, flexural strength, and interlaminar shear strength values, whereas further increases in granite dust content resulted in decreases in the above-mentioned properties. It should be noted that the flexural strain at break gradually grew with an increase in granite dust content up to 5 wt. %.

## Data Availability

No new data were created or analyzed in this study. Data sharing is not applicable to this article.
